# Tylophorine Analog DCB-3503 Inhibited Cyclin D1 Translation through Allosteric Regulation of Heat Shock Cognate Protein 70

**DOI:** 10.1038/srep32832

**Published:** 2016-09-06

**Authors:** Ying Wang, Wing Lam, Shao-Ru Chen, Fu-Lan Guan, Ginger E. Dutchman, Samson Francis, David C. Baker, Yung-Chi Cheng

**Affiliations:** 1 Department of Pharmacology, Yale University School of Medicine, New Haven, CT 06520, USA; 2 State Key Laboratory of Quality Research in Chinese Medicine and Institute of Chinese Medical Sciences, University of Macau, Avenida da Universidade, Taipa, Macao SAR, China; 3 Department of Chemistry, The University of Tennessee, Knoxville, TN 37996, USA.

## Abstract

Tylophorine analog DCB-3503 is a potential anticancer and immunosuppressive agent that suppresses the translation of cellular regulatory proteins, including cyclin D1, at the elongation step. However, the molecular mechanism underlying this phenomenon remains unknown. This study demonstrates that DCB-3503 preferentially binds to heat shock cognate protein 70 (HSC70), which is a determinant for cyclin D1 translation by binding to the 3′-untranslated region (3′ UTR) of its mRNA. DCB-3503 allosterically regulates the ATPase and chaperone activities of HSC70 by promoting ATP hydrolysis in the presence of specific RNA binding motifs (AUUUA) of cyclin D1 mRNA. The suppression of cyclin D1 translation by DCB-3503 is not solely caused by perturbation of the homeostasis of microRNAs, although the microRNA processing complex is dissociated with DCB-3503 treatment. This study highlights a novel regulatory mechanism of protein translation with AUUUA motifs in the 3′ UTR of mRNA by HSC70, and its activity can be allosterically modulated by DCB-3503. DCB-3503 may be used to treat malignancies, such as hepatocellular carcinoma or breast cancer with elevated expression of cyclin D1.

Tylophorine analogs were first identified and isolated from the herbal medicine *Tylophorine indica*^1^, which was originally used to treat asthma and arthritis in India and China, respectively^2^,^3^. Tylophorine analogs exhibit diverse activities against cancer, inflammation, arthritis, lupus, and colitis^4^,^5^,^6^,^7^,^8^,^9^,^10^,^11^. Tylophorine analogs DCB-3503 and *rac*-crytopleurine inhibit global protein synthesis at the elongation step of translation^12^,^13^. This novel activity is distinct from that of other anticancer drugs or protein synthesis inhibitors^12^. Despite the broad range of therapeutic potential offered, only vascular endothelial growth factor receptor 2 (VEGFR2) was reported as the molecular target of tylophorine for its antiangiogenesis activity^9^. Ribonucleoprotein complex containing caprin-1 is associated with tylophorine and responsible for inhibiting translation^14^. We previously reported that structural analogs of tylophorine may not be functional analogs^15^,^16^.

Polypeptide/protein synthesis is directed by mRNA translation in the cytoplasm. Ribosome is associated with mRNA during translation, ensuring correct access of activated tRNAs and containing the necessary enzymatic activities to catalyze peptide bond formation^17^. Translation is precisely controlled, and the translation machinery is functionally converged with several fundamental cell signaling pathways. Abnormally regulated translation may be a major contributor to cancer initiation, drug resistance formation, invasion, and metastasis^17^. Translation is regulated by modulating the level or activity of translation factors, ribosome biogenesis, or small molecule/RNA interactions.

The 70 kDa heat shock proteins (HSP70/HSC70) play fundamental roles in protein homeostasis by chaperoning correct folding, translation, translocation, disaggregation, and degradation^18^,^19^. The highly conserved HSP70/HSC70 share approximately 90% identical sequences in the N-terminus nucleotide binding domain (NBD), but not in the C-terminus substrate binding domain (SBD)^20^. The affinity of SBD to substrate is strictly regulated by NBD through conformational changes induced by the hydrolysis of ATP to ADP^21^. The ADP-binding state exhibits a higher affinity to substrate than the ATP-binding state^21^. Modulating the switch between the ATP- and ADP-binding states controls the chaperone function of HSP70/HSC70^21^.

In the current study, we demonstrate that tylophorine analog, DCB-3503, specifically binds to HSC70. DCB-3503 allosterically regulates ATPase and chaperone activity of HSC70, thus inhibiting the translation of cyclin D1 by promoting binding to the AUUUA motif in the 3′–untranslated region (3′ UTR) of mRNA. This mechanism plays a critical role in the regulation of selected sets of mRNA with specific HSC70 binding motifs by tylophorine analogs, such as DCB-3503.

## Results

### DCB-3503 interacted with HSC70

**Figure 1:**
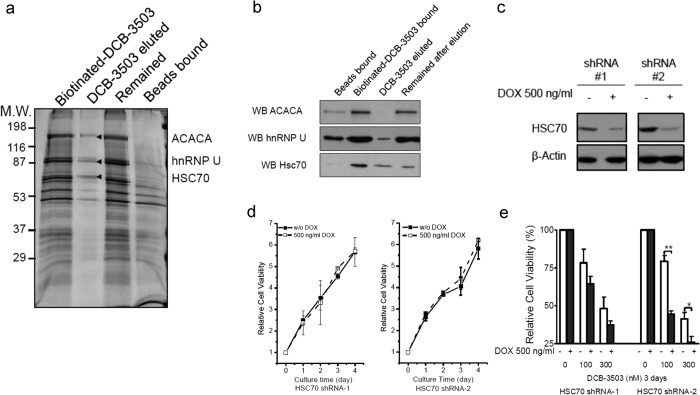
DCB-3503 interacted with HSC70. (**a**) HepG2 lysate was incubated with streptavidin-agarose beads bound with biotinylated-DCB-3503. Bound proteins were eluted and separated by 10% SDS-PAGE. Protein bands were visualized by silver staining, and identified by LTQ Orbitrap mass spectrometry. (**b**) Western blot analysis of acetyl-CoA carboxylase α (ACACA), hnRNP U, and HSC70 as in (**a**). (**c**) Expression level of HSC70 in DOX-inducible HSC70 shRNA RKO cells. Blot of β-Actin served as internal loading control. (**d**) Relative cell viability of RKO cells with DOX-inducible HSC70 shRNA in the presence or absence of DOX. (**e**) Relative cell viability with DCB-3503 treatment in the HSC70-inducible knockdown RKO cells. Results represent at least three independent experiments and are presented as mean ± SD (**p < 0.01; *p < 0.05).

We previously demonstrated that treatment with DCB-3503 especially inhibits the translation of a set of cellular regulatory proteins with relatively short half-lives^12^. Treatment with DCB-3503 shifts the sedimentation profiles of ribosomes and mRNAs toward polysomal fractions^12^. However, the molecular target(s) underlying this novel mechanism of action remains undetermined. We synthesized biotinylated-DCB-3503 to determine the molecular target(s) through affinity purification (The chemical structure of biotinylated-DCB-3503 is shown in the Supplementary Information section in Fig. S1A. An outline of its chemical synthesis and characterization is also given in the Supplementary Information; see Schemes S1 and S2, along with HPLC data in Chart S1). The biotin group is tethered to the 3-position of the phenanthrolizidine system, which is not a determinant of the mode of action of tylophorine analogs (based on a previous structure–activity relationship studies), but reduced cytotoxicity of DCB-3503 (Fig. S1A)^13^. The biotinylated-DCB-3503 affinity–purified complexes were resolved by SDS-PAGE and visualized by silver staining (Fig. 1a,b). Protein bands specifically eluted by DCB-3503 were identified by LTQ Orbitrap mass spectrometry (Fig. 1a). Western blot results showed that acetyl-CoA carboxylase α (ACACA) was eluted by DCB-3503, but ACACA naturally binds to the biotin moiety^22^. hnRNP U bound to the beads and biotinylated-DCB-3503 under the same conditions (Fig. 1b). Among the identified proteins, HSC70 and hnRNP U could be eluted by DCB-3503 (Fig. 1b). Considering the levels of hnRNP U bound to biotylated-DCB-3503 in comparison with the beads alone (Fig. 1b), as well as the previous report that hnRNAP A2/B1 is a molecular target of phenanthrene-based tylophorine derivative-1 (PBT-1)^23^, we considered HSC70 and hnRNP U to be potential molecular targets of DCB-3503 and selected HSC70 for further evaluation.

DCB-3503 treatment did not change the expression of HSC70 in HepG2 and Hela cells (Fig. S1B and S1C). Doxycycline (DOX)-inducible cell lines expressing HSC70 shRNAs were constructed (Fig. 1c); suppression of HSC70 did not affect cell growth for up to 4 days in RKO and HepG2 cells (Fig. 1d and S1D). Total knockdown of HSC70 was lethal (results not shown). Decreased expression of HSC70 did not rescue RKO cells from DCB-3503 treatment but rather increased cell death with DCB-3503 treatment at 100 nM and 300 nM (Fig. 1e).

### Suppression of HSC70 abrogated DCB-3503-inhibited translation of cyclin D1

**Figure 2:**
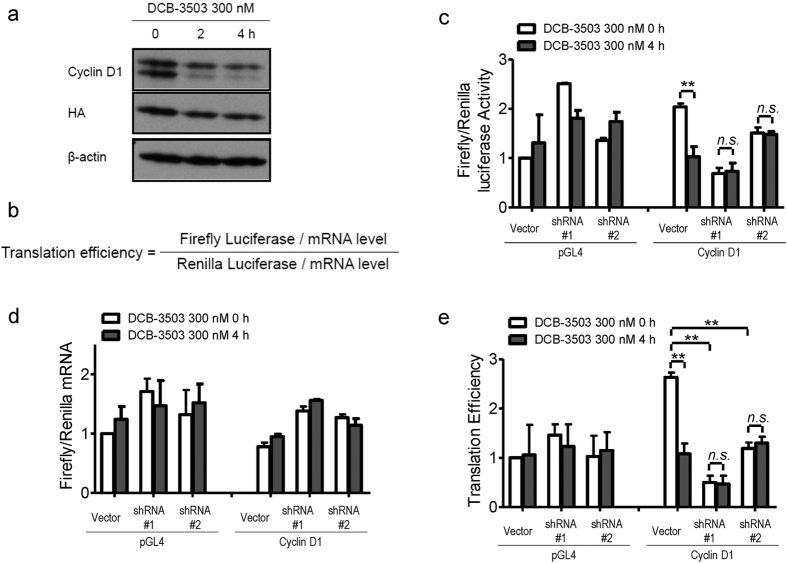
Treatment with DCB-3503 modulated translation efficiency of cyclin D1. (**a**) HepG2 cells were transfected with HA-cyclin D1 expression plasmid for 2 days, and then treated with DCB-3503. Endogenous and exogenous cyclin D1 expression was measured by Western blot analysis. Blot of β-actin served as internal loading control. (**b**) Equation for calculation of translation efficiency. Firefly luciferase reporter with HSC70 binding site on the 3′ UTR of cyclin D1 mRNA (cyclin D1-AUUUA) or vector (pGL4.20) was transiently transfected into RKO cells bearing DOX-inducible HSC70 shRNA together with renilla luciferase plasmid. Two days after transfection, the cells were treated with 300 nM DCB-3503 for 4 h. Relative firefly and renilla luciferase activity (**c**) and mRNA level (**d**) were measured. (**e**) The luciferase values of different constructs with DCB-3503 treatment (**c**) were normalized to mRNA levels (**d**) to obtain translation efficiencies. Results represent at least three independent experiments and are presented as mean ± SD (** *p*<0.01; *n.s.*, nonsignificant).

DCB-3503 treatment inhibits cyclin D1 translation at the elongation step^24^. Translation could be regulated by modulating the activities of initiation factors, sequence-specific RNA-binding proteins, and/or microRNAs targeting the 3′ UTR of mRNA^25^. We constructed the HA-tagged cyclin D1 plasmid by inserting only the open reading frame. The expression of endogenous cyclin D1 was decreased to 10% after 2 h of DCB-3503 treatment (Fig. 2a), whereas HA-tagged cyclin D1 was more resistant to DCB-3503 treatment (Fig. 2a). Considering the long 3′ UTR of cyclin D1 mRNA, we speculate that DCB-3503 treatment might work on the 3′ UTR region to regulate translation of cyclin D1.

We constructed a luciferase reporter bearing the 3′ UTR of cyclin D1 mRNA and transfected it to RKO cells with DOX-inducible HSC70 shRNAs. Renilla plasmid was included in the following transfections to normalize transfection efficiency, extraction conditions, and overall translation status. To distinguish translational output from mRNA turnover, luciferase assays were normalized to the RNA levels of the luciferase-reporter to determine the translation efficiency (defined in Fig. 2b). Treatment with 300 nM DCB-3503 for 4 h inhibited the translation efficiency of the luciferase reporter bearing the 3′ UTR of cyclin D1 mRNA (Fig. 2c–e). Knockdown of HSC70 expression by DOX-inducible shRNAs partially restored the DCB-3503-inhibited translation of cyclin D1 mRNA (Fig. 2c–e). However, the overall translation efficiency significantly reduced with HSC70 suppression compared with vector transfection (Fig. 2e). The translation efficiency of the control pGL4.20 plasmid was changed under the same conditions (Fig. 2c–e).

### Treatment with DCB-3503 induced co-sedimentation of HSC70 and cyclin D1 mRNA on an Optiprep density gradient

**Figure 3:**
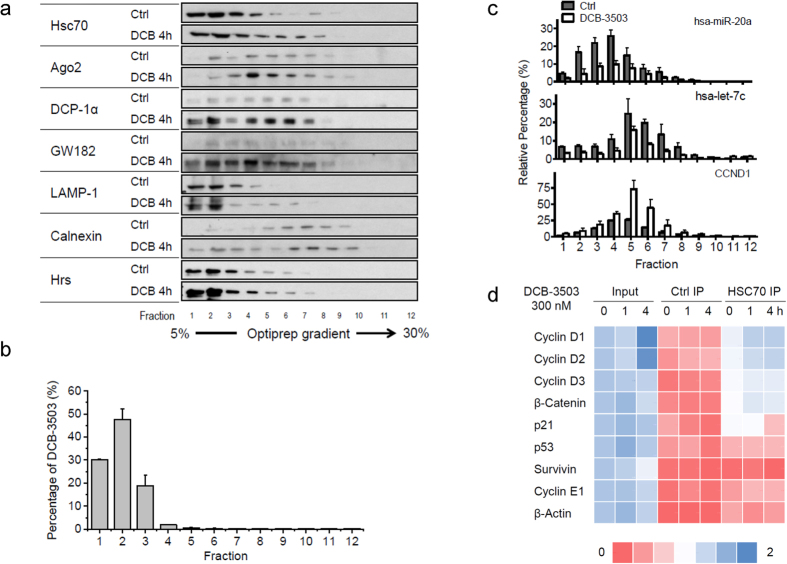
Treatment with DCB-3503 changed the association of HSC70 with miRNA processing complex, miRNAs, and cyclin D1 mRNA. (**a**) Partition of HSC70, Ago2, DCP-1α, GW182, LAMP-1, calnexin, and Hrs from Optiprep gradient fractions obtained from control HepG2 cells or cells treated with 300 nM DCB-3503 for 4 h. (**b**) The relative percentage of DCB-3503 in each fraction was analyzed by LC/MS/MS. (**c**) The relative contents of cyclin D1 mRNA, hsa-miR-20A, and hsa-let-7c in fractions obtained in (**a**) were analyzed using real-time PCR. (**d**) Treatment with DCB-3503 increased the association of cyclin D1, cyclin D2, cyclin D3, and β-catenin mRNAs to HSC70. HSC70-associated mRNA from the control or DCB-3503 treated HepG2 cells was isolated from the HSC70 complex by using RNA-IP method with an HSC70 specific antibody. Quantity of different mRNAs were analyzed by using real-time PCR and normalized to that of the untreated control sample. Results repreesnt at least three independent experiments and are presented as mean ± SD.

HSC70 is a chaperone protein that binds to various substrates^26^. We examined whether DCB-3503 treatment changes the association between HSC70 and specific mRNAs to halt translation. We first determined the association of HSC70 with cellular proteins that regulate translation. Post-nuclear supernatants of the control and DCB-3503-treated HepG2 cells were fractionated on continuous Optiprep iodixanol density gradients (Fig. 3a–c, and S2A). The HSC70 protein in the DCB-3503-treated cells extended from fractions 1–4 to fractions 1–7 (Fig. 3a). DCB-3503 treatment enriched miRNA processing proteins in fractions 2–6, including Argonaut 2 (Ago2), mRNA decapping enzyme DCP-1α, and GW182 (Fig. 3a). The lysosome marker LAMP-1 was localized in fractions 1–3, the endoplasmic reticulum marker calnexin was localized in fractions 6–9, and the multivesicular body marker Hrs was located on the top fraction of the Optiprep density gradients (Fig. 3a). The localization of DCB-3503 in fractions obtained in Fig. 3a was analyzed using LC/MS/MS. DCB-3503 was mainly identified in fractions 1–3 and peaked in fraction 2 (Fig. 3b), suggesting that DCB-3503 was associated with cellular complex/protein(s) rather than in the soluble fractions of the cytosol (fraction 1).

The contents of cyclin D1 mRNA, miR-20a, and let-7c were quantitated through real-time PCR. miR-20a and let-7c from the control, and DCB-3503-treated samples were mainly localized in fractions 2–6 and 5–7, respectively (Fig. 3c). Cyclin D1 mRNA mainly accumulated in fractions 5–7 with DCB-3503 treatment (Fig. 3c), which localized in the same sedimentation fractions as HSC70 with DCB-3503 treatment (Fig. 3a). We thus analyzed whether cyclin D1 is associated with HSC70 through RNA-IP. We selected several genes whose translation was inhibited by DCB-3503 treatment or other tylophorine analogs (cyclin D1, cyclin D2, cyclin D3, β-catenin, p21, p53, survivin, and cyclin E1) from our and others’ work^12^. Among these genes, five repeats of the potential HSC70–binding motifs (AUUUA) can be found in the 3′ UTR of cyclin D1 and cyclin D2 mRNAs, and one repeat in the 3′ UTR of cyclin D3 and β-catenin mRNAs. No potential HSC70-binding sites were identified in the 3′ UTR of p21, p53, survivin, cyclin E1, or β-actin. mRNA of each gene in the HSC70-associated complex was quantitated using real-time PCR and then normalized to that of input from the untreated control. DCB-3503 treatment significantly increased the association of cyclin D1 and cyclin D2 mRNA to HSC70 (Fig. 3d). The association between cyclin D3 and β-catenin mRNA to HSC70 also increased with DCB-3503 treatment (Fig. 3d). However, the mRNA of p21, p53, survivin, cyclin E1, and β-actin did not bind to HSC70 under the same conditions (Fig. 3d). The quantitated result of RNA-IP is shown in Fig. S2B.

### DCB-3503 inhibited translation of cyclin D1 by targeting the HSC70 binding site

**Figure 4:**
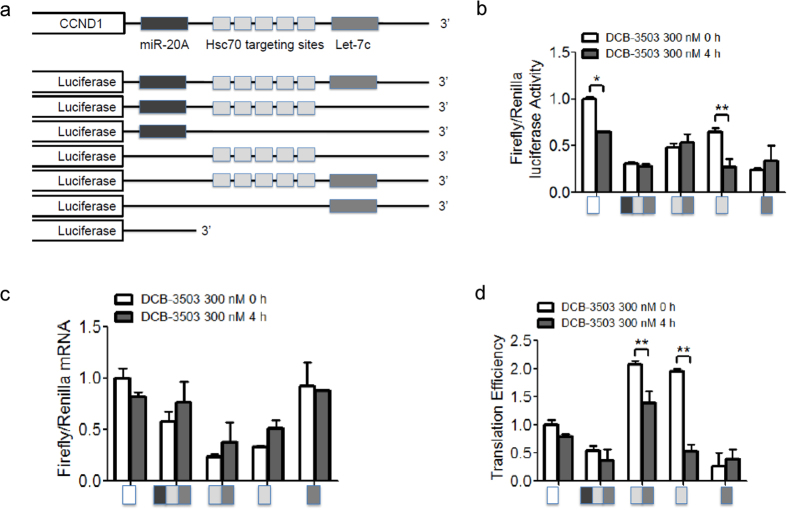
DCB-3503 treatment regulated cyclin D1 translation through HSC70 binding sites. (**a**) Sequence and evolutionary conservation of the miR-20a, let-7c, and HSC70 binding sites in the 3′ UTR of cyclin D1 mRNA. Firefly luciferase reporters with different regions of the 3′ UTR of cyclin D1 mRNA were designed. Hela cells were transiently transfected with different luciferase constructs with different cyclin D1 3′ UTR regions, as shown in (**a**), together with renilla luciferase plasmid. The cells were treated with DCB-3503 2 days after transfection. Relative firefly and renilla luciferase activity (**b**) and their mRNA levels (**c**) were measured. (**d**) The luciferase values of different constructs with DCB-3503 treatment (**b**) were normalized to the mRNA levels (**c**) to obtain translation efficiencies. Results are presented as mean ± SD from at least three independent experiments (**p < 0.01; *p < 0.05).

We analyzed potential regulatory elements in the 3′ UTR of cyclin D1 mRNA. Searching results from TargetScan, miRBase, and mirDB revealed that 3′ UTR of cyclin D1 has targeting sequences for two miRNAs, miR-20a and let-7c. 3′ UTR of cyclin D1 mRNA also has five AUUUA elements, the reported HSC70 binding motif. Therefore, we constructed luciferase reporters with the 3′ UTR of cyclin D1 (nt 1612–3358), miRNA targeting sites, and/or HSC70 binding sites (Fig. 4a).

We transiently transfected luciferase constructs (Fig. 4a) together with renilla plasmid into Hela cells. DCB-3503 treatment for 4 h only decreased firefly luciferase activity in the constructs containing HSC70 binding sites or the vector control (Fig. 4b). Firefly luciferase mRNA increased after DCB-3503 treatment in all constructs, excluding that with a let-7c targeting site (Fig. 4c). The mRNA level of the control firefly luciferase vector reduced after DCB-3503 treatment (Fig. 4c). Normalized results (Fig. 2d) suggested that DCB-3503 treatment significantly decreased the translation efficiency of constructs with HSC70 targeting sites (Fig. 4d). The expression levels of miR-20a, miR-302, and let-7c decreased after DCB-3503 treatment for 1 h (Fig. S2C). The expression levels of miR-369-3p and miR-511, which do not bear targeting sites on the 3′ UTR of cyclin D1 mRNA, remained unchanged under the same conditions (Fig. S2C).

### DCB-3503 treatment dissociated binding of HSC70 with the miRNA processing complex

**Figure 5:**
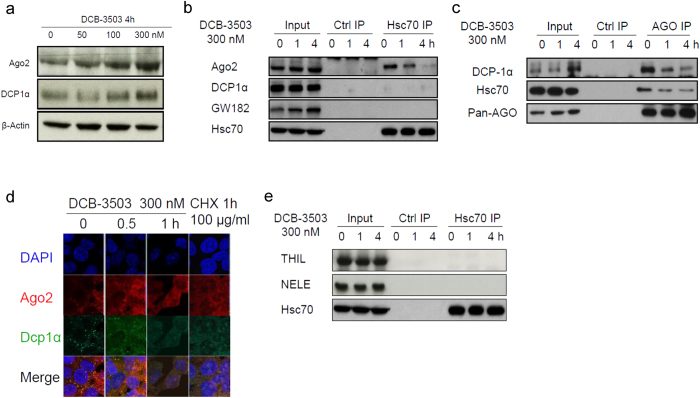
DCB-3503 treatment modulated the association of HSC70 to miRNA processing complex and cyclin D1 mRNA. (**a**) Expression of Ago2 and DCP1α with to 4 h of DCB-3503 treatment in HepG2 cells. (**b**) Association of HSC70 to Ago2, and DCP1α was examined using IP with an HSC70-specific antibody following Western blot in DCB-3503 treated HepG2 cells. (**c**) Association of Ago between HSC70, and DCP1α was examined using IP with pan Ago antibody in DCB-3503 treated HepG2 cells. (**d**) The association of Ago2 and DCP-1α with DCB-3503 treatment or CHX treatment was examined by immunostaining in HepG2 cells. (**e**) Association of HSC70 between TH1L, and NELF-E was examined by immunoprecipitation with HSC70 antibody. Results are from at least three independent experiments.

The 3′ UTR of mRNA is a potent posttranscriptional regulatory element that interacts with mRNA binding proteins and miRNAs^27^. We determined whether DCB-3503 treatment alters the miRNA processing machinery. DCB-3503 treatment for 4 h increased the expression of processing body components (Fig. 5a). Ago2, DCP1α, and GW182 were found to be in the same sedimentation fractions as HSC70 in the DCB-3503-treated HepG2 cells (Fig. 3a). However, DCB-3503 treatment decreased the association between HSC70 and Ago2 in HepG2 cells by using IP with an HSC70-specific antibody (Fig. 5b). HSC70 did not bind to DCP1α or GW182 under the same conditions (Fig. 5b). The amount of HSC70 bound to Pan Ago decreased after DCB-3503 treatment for 4 h in HepG2 cells (Fig. 5c). The binding between Ago and DCP1α also reduced under the same conditions (Fig. 5c). The association of HSC70 with Ago2 also reduced after DCB-3503 treatment in Hela cells (Fig. S3A). The binding between Ago2 and HSC70 was disrupted by RNase treatment (Fig. S3B). The number of microscopically detectable Ago2 and DCP1α complexes decreased in DCB-3503 treatment for 0.5 h, and became undetectable after 1 h (Fig. 5d). Cycloheximide (CHX) treatment that inhibited translation at the elongation step and reduced the microscopically detectable Ago2 and DCP1α complex (Fig. 5d). Microscopically visible miRNA processing bodies in Hela cells also decreased in a time- and dose-dependent manner after DCB-3503 treatment (Fig. S3C–S3E).

We then examined the association between HSC70 and key enzymes that repress RNA polymerase II transcript elongation, including negative elongation factor complex member-C/D (TH1L) and -E (NELF-E)^28^. Immunoprecipitation with HSC70 antibody did not pull down TH1L or NELF-E (Fig. 5e), suggesting that HSC70 was not involved in transcription elongation.

### DCB-3503 allosterically regulated the chaperone activity of HSC70

**Figure 6:**
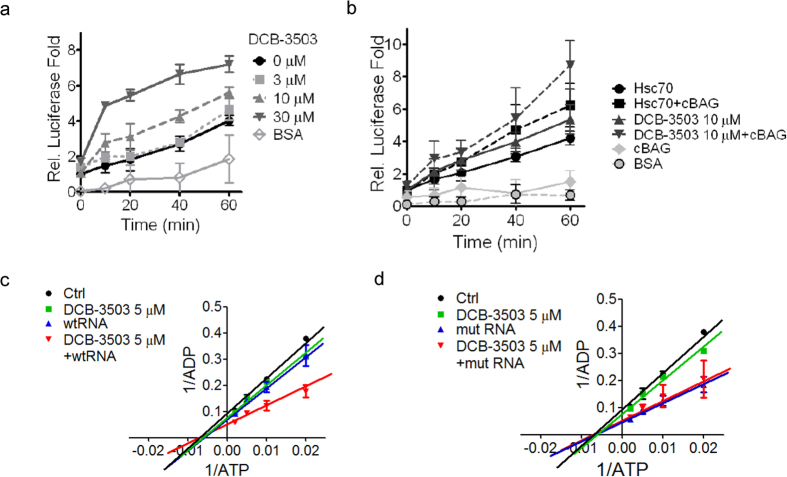
DCB-3503 treatment altered the chaperone activity of HSC70. Chaperone activity of HSC70 in luciferase refolding activity in the presence of (**a**) different concentrations of DCB-3503, and (**b**) cBAG and DCB-3503. Effect of DCB-3503 on the ATPase activity of HSC70 in the presence or absence of (**c**) wtRNA or (**d**) mutRNA. The concentration of generated ADP was analyzed and calculated on the basis of the area under the curve (AUC) on a standard curve calculated with results obtained from the same C18 HPLC column. The *K*_m_ and *V*_max_ values were calculated by fitting the data to the Michaelis-Menten kinetics equation. Results are presented as mean ± SD from at least three independent experiments and.

The ATP or ADP binding state is a determinant for regulating the chaperone activity of HSC70^29^. The chaperone activity of HSC70 was analyzed using the guanidine∙HCl unfolded firefly luciferase method. Increased refolding of luciferase by HSC70 was observed upon adding increasing concentrations of DCB-3503 (Fig. 6a). BSA did not increase refolding of denatured luciferase under the same conditions (Fig. 6a). The C-terminal domain of BAG-1 (cBAG), a co-chaperone of HSC70, stimulated client release from HSC70 by accelerating ADP - ATP exchange^30^. An equal concentration of cBAG increased the refolding of denatured luciferase by HSC70 (Fig. 6b). The combination of cBAG and 10 μM DCB-3503 further enhanced the HSC70-facilitated folding of denatured luciferase (Fig. 6b). The accelerated release of denatured substrate by HSC70 was governed by the switch of ATP to ADP. The ATPase activity of HSC70 was allosterically changed in the presence of DCB-3503 (Fig. 6c–d). The *V*_max_ of HSC70 to ATP increased with the addition of 5 μM DCB-3503, or wild-type RNA (wtRNA) containing the HSC70 binding motif (AUUUA) (Fig. 6c). Combining DCB-3503 and wtRNA further increased the *V*_max_ of HSC70 under the same conditions (Fig. 6c). However, adding of mutant RNA (mutated HSC70 binding site) did not change the *V*_max_ of HSC70 (Fig. 6d). The *K*_m_ of HSC70 was unchanged in the presence of DCB-3503 and/or RNA (Fig. 6c–d).

## Discussion

This study revealed a functional role for HSC70 in the translation of mRNAs having a specific motif. Suppression of HSC70 expression does not affect the viability of cultured cells (Fig. 1d and S1C); however, complete knockout of HSC70 in culture leads to cell death and is embryonically lethal^31^. In addition to other functions of HSC70, DCB-3503 affected the interaction of HSC70 with mRNAs by modulating its ATPase activity. The following model for DCB-3503 inhibitory action against translation is proposed: DCB-3503 binds to HSC70 (Fig. 1a,b) and promotes the ATPase activity of HSC70 (Fig. 6). HSC70 in the ADP binding mode exhibits higher affinity to substrate, for example, the AUUUA motif in the 3′ UTR of cyclin D1 and cyclin D2 mRNA (Figs 3 and 4). Treatment with DCB-3503 increased HSC70-associated cyclin D1 mRNA (Fig. 3a,d) and hence inhibited translation (Fig. 2). The binding of HSC70 to miRNA processing proteins decreased after DCB-3503 treatment, which led to the dissociation of Ago2 and DCP 1α (Fig. 5) and affected miRNA homeostasis. The miR-20a and let-7c binding motifs on cyclin D1 mRNA did not directly regulate translate cyclin D1 translation (Fig. 4). However, we cannot rule out the involvement of other miRNAs in regulating the translation of cyclin D1 mRNA.

Our results opened an interesting therapeutic perspective for regulating the translation of oncogenes by targeting HSC70. HSP70 family proteins share similar N-terminus ATPase and C-terminus chaperone activities^32^. Although HSP70 and HSC70 share approximately 90% homology in the ATPase domain, the binding of HSP70 to mRNA is directed to the native AU-rich element^20^, whereas HSC70 exhibits a high affinity for mRNAs containing AUUUA and U-rich motifs such as Bim and c-fos^19^. Most previous attempts to develop HSC70 inhibitors focused on the ATP binding site, which is a difficult target because of the lack of selectivity among different heat shock proteins^18^,^33^. Our observations support the role of DCB-3503 as an allosteric regulator of HSC70, which differs from existing HSC70 inhibitors.

The regulatory role of DCB-3503 for HSC70 differented from HSC70 inhibitors. Phenylethyynesulfonamide, which binds to the C-terminus of HSP70 but not to that of HSC70 or HSP90, disrupted autophagy but did not induce apoptotic cell death in cancer cells^34^. The immunosuppressive agent 15-deoxyspergualin, which inhibits HSC70 activity by binding to the C-terminus of HSC70 but not to that of HSP70 or HSP90, was approved for the treatment of glomerulonephritis associated with active systemic lupus erythematosus^35^. This agent also exhibits low anti-cancer activity. Apoptozole, a small–molecule specific inhibitor of HSP70 and HSC70, induces apoptotic phenotypes by disrupting the interaction of HSP70 with APAF-1^21^. In consideration of the many opportunities for interference offered by HSC proteins, allosteric regulators that stabilize HSP/HSCs in the ADP-bound substrate binding state have emerged in addition to inhibitors of HSP/HSC ATPase activities. For example, compounds gentamicin and geranylgeranylacetone modulate HSP70 by competing with substrate binding^36^,^37^. Anti-cancer compound MKT-077 selectively bound to the ADP state of HSC70; consequently, HSC70 cannot release its substrate and interferes with the ability of HSC70 to promote cancer cell survival directly^38^,^39^. DCB-3503 enhanced the ATPase activity of HSC70 especially in the presence of the “AUUUA” motif (Fig. 6), and increased binding between HSC70 and “AUUUA”-containing mRNAs (Fig. 3 and S2), thus inhibiting the translation of this set of mRNAs. Halting the translation of mRNAs containing the “AUUUA” motif that governs cell cycle checkpoints is essential to cancer cell survival^40^. Aside from inhibiting the translation to “AUUUA”-containing mRNAs, DCB-3503 inhibits the synthesis of proteins with relatively short half-lives^12^. Therefore, it is also reasonable that knockdown of HSC70 enhanced cancer cell growth after DCB-3503 treatment (Fig. 1d).

Cyclins are a group of regulators of cyclin-dependent kinase (CDK) that coordinate to control shuffle of during mitotic events^41^. Cyclin D1 forms a complex with CDK4 or CDK6, whose activity governs the transition of the G1/S phase of the cell cycle^41^. About 17% of all breast cancers tested were associated with elevated expression of cyclin D1^42^. The expression of cyclin D1 is primarily regulated by Ras-mediated signaling pathways during transition to the G1 phase^43^. Nucleus cyclin D1 is phosphorylated at the T286 residue, exported from the nucleus, and then degraded via the ubiquitin-mediated proteolysis pathway at the end of the S phase^43^.

Translation of cyclin D1 can be controlled by miRNAs. For instance, miRNA-193b suppresses cyclin D1 by binding to its 3′ UTR in melanoma Malme-3M cells^44^. The expression of miR-21 induces translation of the cyclin D1 in normal hepatocytes by activating the mammalian target of rapamycin complex 1 (mTORC1)^45^. The miRNA-mediated regulation of translation is controlled by the Ago2/DCP1α complex^46^. The phosphorylation of Ago2 is sufficient to modulate the binding of mature miRNA to target mRNAs^47^. The mRNA decapping enzyme DCP1α plays a fundamental role in the miRNA-mediated suppression of translation^48^. miRNA-mediated gene silencing activity is usually coupled with the elevated expression of DCP1α^49^. However, Ago2 and DCP1α decreased microscopically visible association following treatment with DCB-3503 or CHX (Fig. 5d and S3). This phenomenon was also observed in a previous report that showed miRNA mediates the upregulation of translation^50^. Our results suggested that DCB-3503 treatment decreased the association between Ago2 and DCP1α, even though theirs was increased (Fig. 5). Although the expression of miRA-20a and let-7c was decreased (Fig. S2C), DCB-3503 treatment did not affect the translation efficiency of luciferase constructs bearing only miR-20a or let-7c targeting sites (Fig. 4). These results suggest that miRNAs were not crucial to the DCB-3503-inhibited translation of cyclin D1 (Figs 4 and 5). However, the association between HSC70 and Ago2 suggests HSC70 may govern miRNA homeostasis through association with processing body components. DCB-3503 treatment led to the dissociation of Ago2 from HSC70 and DCP1α (Fig. 5), which also suggests that HSC70 is responsible for the translation of a specific subset of mRNAs with certain motif(s) and/or miRNA homeostasis. HSC70 was not associated with transcription complexes under DCB-3503 treatment (Fig. 5e), suggesting the specific regulation of translation. We could not rule out the possibility that other miRNAs and Ago2 are involved in the regulation of cyclin D1 translation with DCB-3503 treatment.

Many laboratories including ours, have attempted to identify the molecular targets of tylophorine analogs. Saraswati *et al*. reported that tylophorine targets VEGFR2 to exert antiangiogenic and antitumor activities^9^. The authors suggested that tylophorine located at the ATP-binding sites of the VEGFR2 kinase domain and competes with cellular ATP through molecular docking stimulation^9^. However, this study did not show any direct physical interaction between tylophorine and VEGFR2. The structure of tylophorine bound to VEGFR2 is the same as that of (−)-R-tylophorine^16^. Tylophorine or (−)-R-tylophorine exhibits similar cytotoxicity against HepG2, PANC-1, and CEM cells^16^. The sensitivity to NF-κB, CRE, and AP-1 signaling pathways is much lower than DCB-3503 and its functional analogs^16^. The allosteric modulation of the ATPase activity of HSC70 by DCB-3503 (Fig. 6) also differed from the inhibition effect of tylophorine on VEGFR2^9^.

Qiu *et al*. identified a ribonucleoprotein complex containing caprin-1 as the molecular target of tylophorine to suppress tumor growth^14^. PBT-1, the tylophorine analog used in the study is a dibenzoquinoline derivative that lacks the important E ring directly related to activity and mode of action^15^,^16^. In addition, the D ring of tylophorine to which biotin moiety was added may change the function of parental compounds on the basis of our previous structure–activity relationship study^15^,^16^. Both Lee *et al*. and our group have demonstrated that the size of the fifth ring on the backbone of tylophorine analogs is a determinant for the mode of action^13^,^51^. Chen *et al*. determined that HSP90 and hnRNP A2/B1 bind to tylophorine analogs using a biotinylated phenanthrene-based tylophorine derivative (PBT-1-6L) through chemical proteomic methods^23^. The fourth ring on PBT-1-6L was open, which differed from the intact fourth ring of DCB-3503 we used in the current study. We have demonstrated that PBT analogs and DCB-3503 exhibit different modes of action^16^. The cytotoxicity of PBT analogs were in the micromolar range, whereas DCB-3503 analogs exhibited nanomolar IC_50_ under the same conditions. Our results also demonstrated that PBT analogs lost selectivity against key signaling pathways, including NF-κB, CRE, AP-1, and GRE; meanwhile DCB-3503 potently inhibited the NF-κB signaling pathway with nanomolar concentration. In addition, the CH_3_O- moiety on the R3 and R4 positions are required to maintain the activity of tylophorine analogs *in vivo*^15^. Basing from the above structure–activity relationship study, we considered these tylophorine analogs bearing different modes of action compared with DCB-3503 series of tylophorine analogs^15^,^16^. Therefore, these different groups of tylophorine analogs bear different molecular targets.

Hepatocellular carcinoma (HCC), a highly chemo-resistant cancer, is the fifth most common cancer worldwide and the second leading cause of cancer-related deathes in China^52^. HCC patients are most often diagnosed at advanced stages with limited treatment. Sorafenib (Nexavar^®^), a small-molecule inhibitor of tyrosine kinases associated with Raf and vascular endothelial growth factor receptors, remains the only FDA approved drug to treat HCC since 2007^53^. However, sorafenib’s activity is limited because of the high resistance rate of HCC and significant side effects^54^. Cyclin D1 amplification occurred in about 5–8% of all HCC patients and 16–22% in breast cancer patients in TCGA databases (http://www.cbioportal.org/index.do, accessed on January 10, 2016). Therefore, the novel allosteric regulation of cyclin D1 translation by DCB-3503 may be used alone or in combination for the treatment of HCC or breast cancer with cyclin D1 amplification.

In summary, DCB-3503 inhibited the translation of cyclin D1 by allosterically regulating the ATPase activity of HSC70. Its effect on translation is distinct based on different motifs in the 3′ UTR of mRNA. The binding mode between DCB-3503 and HSC70 is different from existing HSC70 inhibitors. We propose DCB-3503 as a potential lead compound for developing specific translation inhibitor-based unique mRNA sequences. The underlying mechanisms for regulating the translation of mRNAs without the “AUUUA” motif by DCB-3503 remain unexplored in the current study. The question of whether or not hnRNP U that binds to DCB-3503 serves as the molecular target regulating the translation of other mRNAs should be studied further.

## Materials and Methods

### Materials

Cell culture media, fetal bovine serum (FBS) were purchased from Invitrogen. The DCB series of compounds were synthesized in Dr. David Baker’s laboratory at University of Tennessee. Doxycycline was purchased from Sigma–Aldrich (St. Louis, MO).

### Affinity purification

The affinity purification method was adopted from the reported by Emami *et al*.^55^. Cells were lysed in protein-binding buffer (PBB, 20Mm HEPES, pH 7.9, 100 mM NaCl, 0.5 mM EDTA, 0.5% Nonidet P-40, 6 mM MgCl_2_, 5 mM 2-mercaptoethanol, complete protease inhibitor). Biotinylated-DCB-3503 was bound overnight at room temperature to a slurry of 50% streptavidin-agarose beads (Invitrogen) in buffer containing 50% DMSO and 50% PBB. Beads were washed to remove unbound compound and then incubated with pre-cleared whole-cell lysates or recombinant protein in 2.5% BSA. Bound proteins were eluted with DCB-3503. Proteins that remained bound to beads were eluted with SDS loading buffer. Samples were separated with SDS-PAGE, and examined by silver staining or Western blot. Specific bands from silver-stained gel were analyzed by LTQ Orbitrap mass spectrophotometry (Yale University W.M. Keck Foundation Biotechnology Resource Laboratory).

### Statistical analysis

Data were analyzed by two-tailed Student’s *t*-test (Microsoft Office Excel and Graphpad software). The difference was considered to be statistically significant when *p* < 0.05.

## Additional Information

**How to cite this article**: Wang, Y. *et al*. Tylophorine Analog DCB-3503 Inhibited Cyclin D1 Translation through Allosteric Regulation of Heat Shock Cognate Protein 70. *Sci. Rep.* **6**, 32832; doi: 10.1038/srep32832 (2016).

## Supplementary Material

Supplementary Information
