# Supplementary material for: Tylophorine Analog DCB-3503 Inhibited Cyclin D1 Translation through Allosteric Regulation of Heat Shock Cognate Protein 70
Source: Sci Rep. 2016 Sep 6;6:32832. doi: 10.1038/srep32832 (PMC5011780; doi:10.1038/srep32832)
Supplement: Supplementary Information [file srep32832-s1.pdf]

Supplementary Information

Tylophorine Analog DCB-3503 Inhibited Cyclin D1 Translation through Allosteric Regulation of Heat Shock Cognate Protein 70

Ying Wang, Wing Lam, Shao-Ru Chen, Fu-Lan Guan,

Ginger E. Dutchman, Samson Francis, David C. Baker, Yung-Chi Cheng

Chemical synthesis of biotinylated DCB-3503

Quantitative structure–activity relationship (QSAR) studies with a number of DCB-3503 analogues using comparative molecular field analysis (CoMFA®, Tripos Associates) led to the conclusion that the 3-position of these phenanthrolizidine systems was most suited for tethering the biotin moiety for immobilization of a probe via the classical streptavidin procedure. Indeed, modeling a biotin-tethered probe with a suitably long tether, as in compound **13** (Scheme S2), with streptavidin indicated that the pharmacophore remained exposed. The strategy for the synthesis of **13** made use of triazole "click chemistry," whereby two fragments, the alkyne intermediate **11** and the biotin-tethered azido compound **12**, were conjugated via a 1,4-substituted 1,2,3-triazole to give **13**.

Synthesis of the requisite alkyne intermediate **11** (see Scheme S1) proceeded from the 3-hydroxy intermediate **1** by propargylation to give the propargyl derivative **2.** Selective reduction of the CO_2Me group of **2** by $LiAlH_4$ at ice-bath temperatures gave the alcohol **3**. Conversion to the benzylic bromide **4** was achieved by PBr_3. N-Alkylation of the amino acid fragment **5** was then achieved by reaction of **4** with **5** in DMF with K_2CO_3 to give **6**. Internal amidation with AcOH in MeOH, followed by ester hydrolysis in KOH/dioxane and subsequent acidification, then furnished the cyclic ketocarboxylic acid **8**. Friedel–Crafts cyclization under the agency of triflic anhydride then gave the ketoamide **9**, which was selectively reduced with K-Selectride® to a single diastereomeric alcohol **10**. Reduction of the cyclic amide with $LiAlH_4$ at −10 °C gave the requisite **11**.

"Click chemistry" of the biotin-tethered azide **12** (see Scheme S2) and the alkyne **11** furnished the required biotin-tethered DCB-3503 **13**. Analysis of the product by HPLC showed a product of ~95% purity (see Chart S1); MALDI-TOF MS: Calcd. for $C_{44}H_{59}N_7O_{10}S$, *m/z* 877.404; M^+ + Na, *m/z* 900.394; Found, *m/z* 900.410. LC–MS analysis by TurboSpray MS gave M^++ H, *m/z* 878.5. Details of the synthesis of **13** and related compounds will be reported elsewhere.

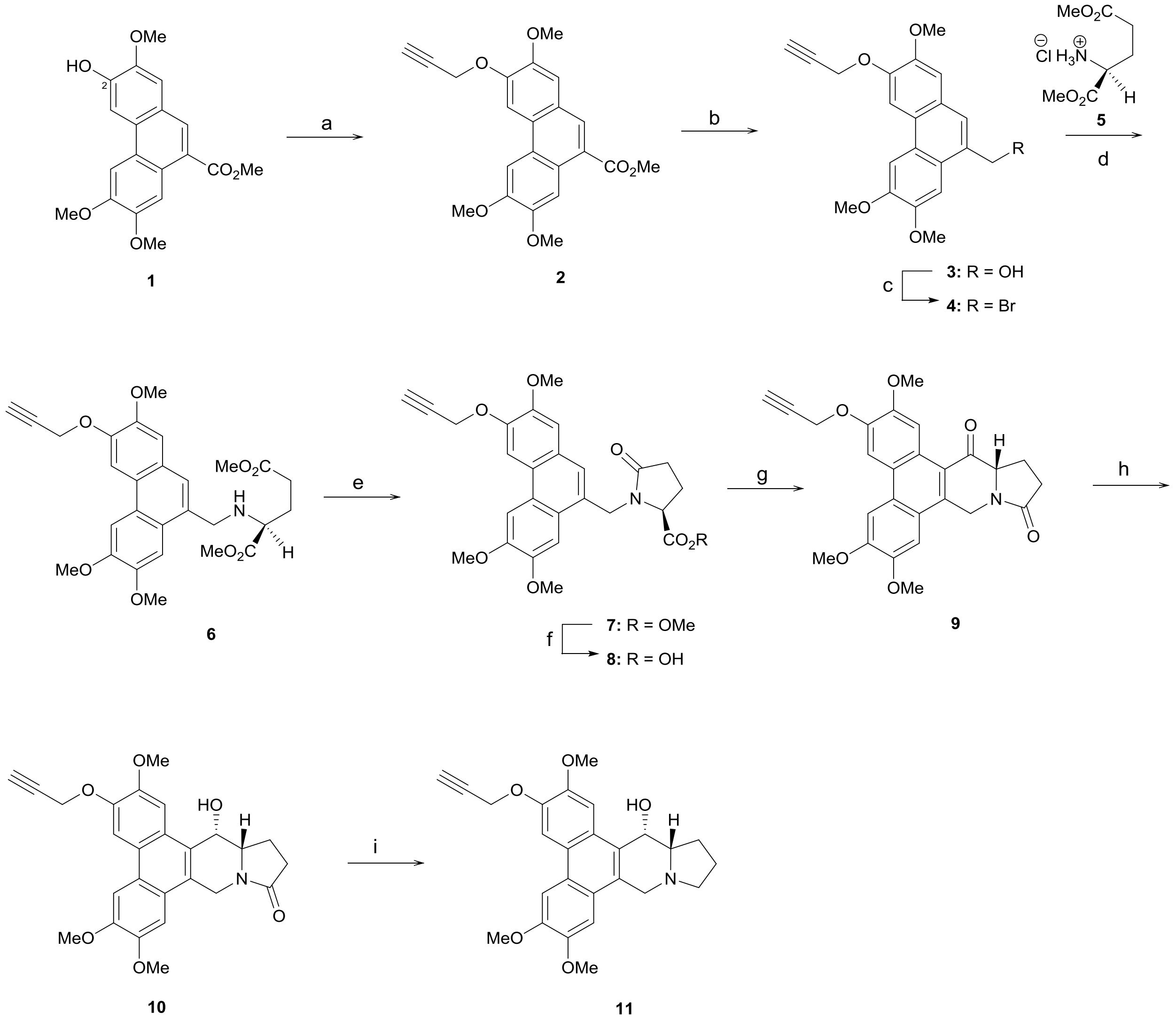

Scheme S1. Synthesis of compound **11**. Reagents and conditions: (a) propargyl bromide, NaH, DMF, 0 °C, 24 h; (b) $LiAlH_4$, THF, 0 °C, 5 h; (c) PBr_3, $CHCl_3$, 0 °C; (d) **5**, K_2CO_3, DMF, 24 h; (e) AcOH, MeOH, 3 h; (f) (1) 2 N KOH, 1,4-dioxane, MeOH; (2) H_3O^+ (g) Tf_2O, CH_2Cl_2, -30 °C, 48 h; (h) K-Selectride®, THF, -78 °C; (i) $LiAlH_4$, THF, -10 °C, 4 h.

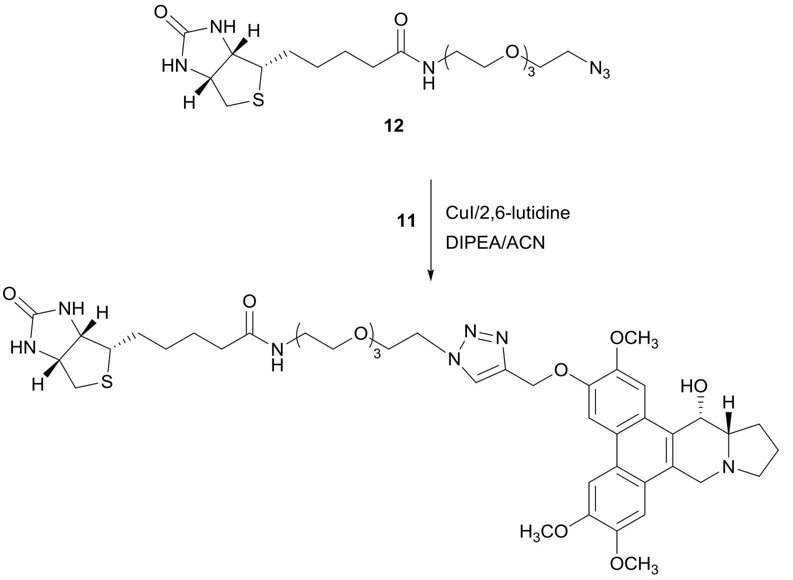

Biotin tethered DCB-3503 (**13**)

Scheme S2. Synthesis of biotinylated DCB-3503 (**13**).

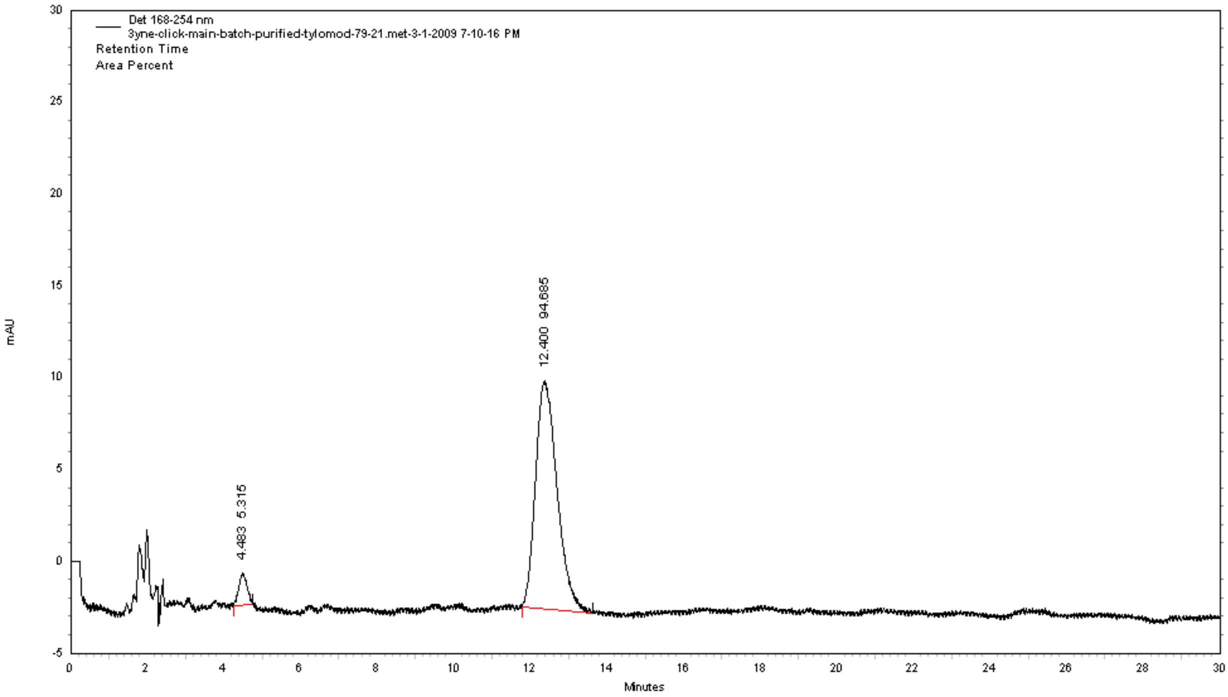

Chart S1. HPLC Trace of biotinylated DCB-3503 (**13**) which elutes at 12.4 min. with ~95% purity.

Supplementary materials and methods

Cell lines, growth conditions, and establishment of a doxycycline-regulated RKO cell line with inducible expression system

HepG2 (human hepatocellular carcinoma) and RKO (human colon carcinoma) cells were maintained in RPMI 1640 supplied with 10% FBS. Hela (human cervical carcinoma) cells were maintained in DMEM medium supplied with 10% FBS.

Stable RKO cell lines harboring pcDNA6/TR was selected by 5 µg/ml blasticidin (InvivoGen, San Diego, CA). The pENTR/H1/TO vector expressing shRNAs were transfected into permanent RKO-pcDNA6/TR cells. Clones were selected using 0.1 mg/ml Zeoicn (Invitrogen). Doxycycline (DOX) was used to induce shRNA expression. The level of knock down of protein and the homogeneity of clones were determined by Western blotting and confocal microscopy, respectively. Clones with homologous expression of shRNAs were isolated and expanded.

Luciferase Reporter, shRNA, and Plasmids

For luciferase reporter constructs, oligonucleotides bearing four repeats of HSC70 binding site, and predicted hsa-miR-20a and hsa-let-7c binding sites (Figure 4A) were cloned into pGL4.20 vector (Promega). Oligonucleotide sequences are provided in Table S1.

The shRNAs for downregulation of HSC70 were designed by the software provided by Invitrogen. The complementary DNA oligonucleotides were: HSC70 shRNA-1 (5'-GCTGTTGTCCAGTCTGATATGcgaaCATATCAGACTGGACAACAGC-3')

(SENSE-loop-ANTISENSE), HSC70 shRNA-2 (5’-GCCCAAGGTCCAAGTAGAATAcgaaTATTCTACTTGGACCTTGGGC-3’) (SENSE-loop-ANTISENSE).

RNA and miRNA isolation, real-time PCR

Total RNA was isolated by Trizal® (Invitrogen). cDNA was synthesized from total RNA using random primer according to the protocol of iScript cDNA synthesis kit (Bio-Rad Laboratories, Hercules, CA), and expression level of specific genes was quantitated by real-time PCR using the SsoFastTM EvaGreen supermix (Bio-Rad Laboratories). The level of β-actin was used as internal control. Expression levels of microRNAs were quantified by the TaqMan® microRNA Assays (Applied Biosystems, Foster City, CA). The level of RNU-6B was used as internal control.

Antibodies

The following antibodies were used: rabbit and mouse IgG, anti-Hrs mAb (ab56468, Abcam), anti-Ago2 polyclone (07-590, Millipore), anti-DCP1α polyclone (a gift from Dr. J. Lykke-Andersen, University of Colorado), anti-GW182 (a gift from Dr. Marvin J Fritzler, Alberta Research and Innovation Authority), anti-HSC70 (sc-7298, Santa Cruz; SPA-815, Stressgen), anti-Lamp-1 (ab25630, Abcam), anti-Calnexin (2679, Cell Signaling), and anti-β-actin (Sigma–Aldrich). For western blots all antibodies were used at a 1:2,000 dilution, except for anti- DCP1α (1:10,000), and anti-Lamp-1 (1:100). For immunofluorescence anti-Ago2, anti-DCP1α antibodies were used at 1:1,000

dilution.

OptiPrep iodixanol continuous density gradient centrifugation

OptiPrep iodixanol density media was obtained from Sigma–Aldrich. Continuous density gradient centrifugation was performed as described previously [5]. Briefly, 10^8 HepG2 cells were washed three times in ice-cold PBS and resuspended in 1 ml hypotonic buffer containing 0.25 M sucrose, 4 mM $MgCl_2$, 8.4 mM $CaCl_2$, 10 mM EDTA, 50 mM HEPES–NaOH at pH 7.0 with Complete Mini Protease Inhibitor (Roche Scientific), 4% ribonucleoside vanadyl (New England Biolabs), and 400 U RNAse Out (Invitrogen). Cells were lysed by 30 strokes of passages through a 20-gauge needle, and centrifuged at 1,000 g for 5 minutes for three times to remove cellular debris and nuclei. Continuous 5–30% Optiprep iodixanol gradients were prepared the previous day in 78 mM KCl, 4 mM $MgCl_2$, 8.4 mM $CaCl_2$, 10 mM EDTA, and 50 mM HEPES-NaOH at pH 7.0 using a gradient mixer and stored overnight at 4 °C. Post-nuclear supernatant was centrifuged at 36,000 rpm at 4°C using a SW 41Ti Beckman Coulter rotor for 80 minutes. Each sample was divided into 12 fractions and collected by ISCO density gradient fractionation system (ISCO).

Liquid chromatography-mass spectrometry (LC-MS)

Proteins in each fraction obtained from the OptiPrep iodixanol gradient centrifugation were removed by acetonitrile:methanol (2:1) precipitation. The aqueous phase was dried, and reconstituted to 100 µl using acetonitrile:water solution (12:88). The

chromatographic separation was achieved on a ZORBAX 3.5 µM extended-C_{18} column (2.1 × 50 mm, Agilent, Palo Alto, CA) on Agilent 1200 HPLC series (Agilent). The ionized mass was detected by Applied Biosystems Sciex 4000 Q-trap® mass spectrometer (Applied Biosystems). Data acquisition was carried out by Analyst 1.4.2® software. Quantification was performed using multiple reaction monitoring (MRM) of the transitions of *m/z* 410 to *m/z* 392 for DCB-3503.

Immunofluorescence

Laser-scanning confocal immunofluorescence imaging was performed using an inverted Zeiss 710 Duo Confocal microscope. Briefly, cells were fixed with 4% paraformaldehyde, permeabilized with 0.5% Triton X-100 in PBS, blocked by 3% BSA. The cells were then incubated with optimized dilution of primary antibodies overnight, and followed by fluorescent conjugated secondary antibody in 1%BSA for 1 hour at room temperature. After washing with PBS for three times, the cells were saved in ProLong® Gold antifade-reagent (Life Technologies).

Immunoprecipitation of HSC70 associated protein and mRNA complexes (RNA-IP)

RNA-IP was performed as described previously [6]. Briefly, HepG2 cells were lysed by polysome lysis buffer (100 mM KCl, 5 mM $MgCl_2$, 10 mM HEPES, pH 7.0, 0.5% NP 40, 1 mM DTT, 100 U/ml RNase inhibitor, 2 mM vanadyl ribonucleoside complexes solution, protease inhibitor cocktail). Supernatant was immunoprecipitated with

prebound biotin-conjugated anti-HSC70 antibody (Enzo Life Sciences, ADI-SPA-815B), and Dynabeads® MyOne™ Streptavidin T1 beads at 4°C overnight. Beads were washed by NT 2 buffer (50 mM Tris-HCl, pH 7.4, 150 mM NaCl, 1 mM $MgCl_2$, 0.05% NP-40) for 4 times before addition of SDS loading buffer for protein analysis or treated with 30 μg proteinase K in NT2 buffer (50°C for 1 hour) for isolation of RNA. HSC70-associated RNA was isolated by phenol–chloroform extraction and glycogen–sodium acetate–ethanol participation. RNA bound to HSC70 was analyzed by real-time PCR. Biotin conjugated Rat-IgG1 (Alpha Diagnostic International, 200005-11-B) was used as isotype control.

Characterization of ATP hydrolysis activity of HSC70

Characterization of the ATP hydrolysis activity of HSC70 was described previously [7]. Briefly, 0.25 μM Hsc70 was incubated in the presence or absence of various concentrations of compound, ATP, and 0.5 μM RNA in 50 μl reaction buffer (25 mM MOPS, pH 7.4, 10 mM $Mg(OAc)_2$, 30 mM KOAc, 2 mM DTT, 1 mg/ml BSA) at 37°C for 10 minutes. Synthetic wide type (wt) RNA (5'-UCCCAGUUGGAUUUAUCCAGCUGG-3') and its mutant form (5'-UCCAGUUGGAgcaAUCCAGCUGG-3') were added to the reaction as indicated on Figure 6A&6B. The reaction was stopped by adding 45% trichloroacetic acid to a final concentration of 15% (v/v). The aqueous phase was washed with 1,1,2-trichlorotrifluoroethane: trioctylamine (55:45, v/v) twice. The concentrations of ATP and ADP in the aqueous phase were analyzed by HPLC.

Expression and purification of recombinant HSC70 and cBAG

Primers for the construction of expression vectors of His-tagged full-length Hsc70, cBAG, and firefly luciferase are shown in Table S1. Recombinant proteins were overproduced in *E.coli* BL21 (DE3) pLysS cells (Promega, Madison, WI) at 22°C for 4 hours, and purified by Ni^{2+}-NTA agarose [8].

Refolding of denatured luciferase

Recombinant firefly luciferase was dissolved in 6 M guanidine·HCl, 100 mM Tris·HCl, 1 mM DTT, pH 7.7 [9]. The refolding reaction was started by addition of 2 µl denatured luciferase (0.1 µM) to refolding buffer with a final dilution factor of 1:100. DCB-3503 and/or cBAG were added at the beginning of the assay. Luciferase activity was measured for the time indicated on Figure 6A&6B for a total of 60 minutes. Equal concentration of BSA was used as a negative control.

Supplementary Figure Legends

Figure S1. (A) Chemical structure of DCB-3503 and biotinylated-DCB-3503, together with IC_{50} against HepG2 cells for 24 hours. The expression level of HSC70 with the treatment of DCB-3503 in (B) HepG2 cells and (C) Hela cells. (D) Relative cell viability of HepG2 cells transiently transfected with HSC70 shRNAs. Results are representative of at least three independent experiments and presented as mean ± SD.

Figure S2. (A) Density of the OptiPrep gradient fractions from samples presented in Figure 3A–3C. (B) Normalized level of different mRNAs associated with HSC70 with the treatment of DCB03503 in HepG2 cells detected by real-time PCR. (C) The expression level of selected miRNAs with the treatment of DCB-3503 in HepG2 cells. Results are representative of at least three independent experiments and presented as mean ± SD.

Figure S3. (A) Association of HSC70 between Ago2 was examined by immunoprecipitation with HSC70 specific antibody following Western blotting in DCB-3503-treated Hela cells. (B) The HSC70-associated complex was treated with 100 μg/ml RNase for 30 minutes before being subjected to SDS-PAGE and immunoblotting of Ago 2. (C) The association of Ago2 and DCP-1α with the treatment of different concentrations of DCB-3503 or CHX was examined by an immunostaining method in HepG2 cells. The association of Ago2 and DCP-1α with the treatment of different (D) time and (E) concentrations of DCB-3503 or CHX in Hela cells was examined by an immunostaining method. Results are representative of at least three

independent experiments.

Table S 1. Primers used for plasmid constructions.

	Forward Primer (5'-3')	Reverse Primer (5'-3')	Vector
Cyclin D1-3'UTR	AAATATATCTCGAGGGGAATCCTTTGGTGCCAACTGGTGT	AAATATATGCGGCCGCCCTTGCACCCATGCCTGTCCA	pGL4.20
Cyclin D1-20a-AUUUA	AAATATATCTCGAGGGGAATCCTTTGGTGCCAACTGGTGT	AAATATATGGATCCTGTCTGCCCGCCAAAGCAGG	pGL4.20
Cyclin D1-AUUUA	AAATATATTCTAGACCTGATAAAGCACAGCTGTA	AAATATATGGATCCTGTCTGCCCGCCAAAGCAGG	pGL4.20
Cyclin D1-let-7c	AAATATATTCTAGAGTACTAGTTTTAGTTTTCTCT	AAATATATGCGGCCGCCCTTGCACCCATGCCTGTCCA	pGL4.20
HA-cyclin D1	AAATATATGGATCCATGGAACACCAGCTCCTGTGCTGCG	AAATATATCTCGAGTTAGGCGTAGTCAGGCACGTCGTAAGGATAGATGTCCACGTCCCGCACGTCGG	pcDNA5/TO
His-HSC70	AAATATATCATATGATGTCCAAGGGACCTGCAGTTGGTA	AAATATATCTCGAGATCATTAATCAACCTCTTCAATGGTGGGC	pET28a
His-cBAG	AAATATATCATATGATGAACAGTCCACAGGAAGAGGT	AAATATATCTCGAGTCACTCAGTCTCCTGGCAGATGT	pET28a

Table S2. Primers used for qPCR analysis.

	**Forward primer (5' – 3')**	**Reverse primer (5' – 3')**
Cyclin D1	TTCGTGGCCTCTAAGATGAAGG	GAGCAGCTCCATTTGCAGC
Cyclin D2	ACCTTCCGCAGTGCTCCTA	CCCAGCCAAGAAACGGTCC
Cyclin D3	TACCCGCCATCCATGATCG	AGGCAGTCCACTTCAGTGC
β-Catenin	AACCTTTCCCATCATCGTGAG	TGAACCAAGCATTTTCACCAG
p21	TGTCCGTCAGAACCCATGC	AAAGTCGAAGTTCCATCGCTC
p53	CAGCACATGACGGAGGTTGT	CCAGACCATCGCTATCTGAGC
Survivin	AGGACCACCGCATCTCTACAT	AAGTCTGGCTCGTTCTCAGTG
Cyclin E1	GCCAGCCTTGGGACAATAATG	AGTTTGGGTAAACCCGGTCAT
β-Actin	ATTGCCGACAGGATGCAGAA	GCTGATCCACATCTGCTGGAA

References

1 Furstner, A. & Kennedy, J. W. Total syntheses of the tylophora alkaloids cryptopleurine, (-)-antofine, (-)-tylophorine, and (-)-ficuseptine C. *Chemistry* **12,** 7398-7410, (2006).

2 Seidel, G. & Furstner, A. Suzuki reactions of extended scope: the '9-MeO-9-BBN variant' as a complementary format for cross-coupling. *Chem Commun (Camb)* **48,** 2055-2070, (2012).

3 Mamane, V., Hannen, P. & Furstner, A. Synthesis of phenanthrenes and polycyclic heteroarenes by transition-metal catalyzed cycloisomerization reactions. *Chemistry* **10,** 4556-4575, (2004).

4 Han, S. Y., Park, S. S., Lee, W. G., Min, Y. K. & Kim, B. T. Synthesis of a novel biotin-tagged photoaffinity probe for VEGF receptor tyrosine kinases. *Bioorg Med Chem Lett* **16,** 129-133, (2006).

5 Mazumdar, B. *et al.* Hepatitis C virus infection upregulates CD55 expression on the hepatocyte surface and promotes association with virus particles. *J Virol* **87,** 7902-7910, (2013).

6 Ozer, A. *et al.* Quantitative assessment of RNA-protein interactions with high-throughput sequencing-RNA affinity profiling. *Nat Protoc* **10,** 1212-1233, (2015).

7 Yamagishi, N., Ishihara, K. & Hatayama, T. Hsp105alpha suppresses Hsc70 chaperone activity by inhibiting Hsc70 ATPase activity. *J Biol Chem* **279,** 41727-41733, (2004).

8 Jinwal, U. K. *et al.* Hsc70 rapidly engages tau after microtubule destabilization. *J Biol Chem* **285,** 16798-16805, (2010).

9 Han, W. & Christen, P. Mutations in the interdomain linker region of DnaK abolish the chaperone action of the DnaK/DnaJ/GrpE system. *FEBS Lett* **497,** 55-58, (2001).

Figure S1

a

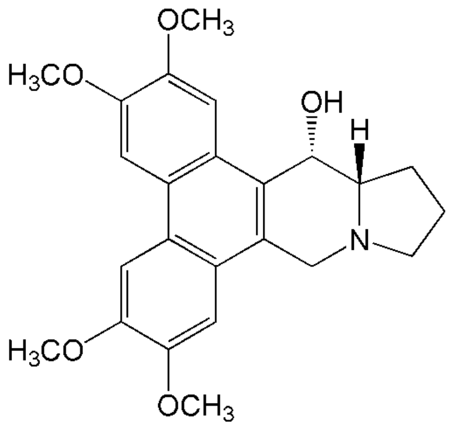

DCB-3503 (NSC-716802)
HepG2 IC_{50}=300±19 nM (24 h)

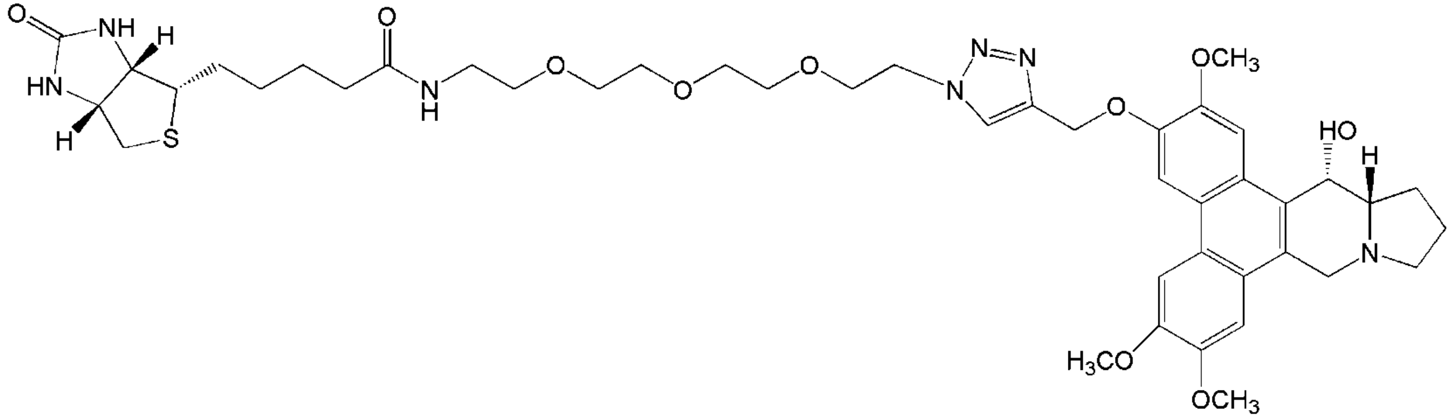

Biotinylated-DCB-3503
HepG2 IC_{50}>500 nM (24 h)

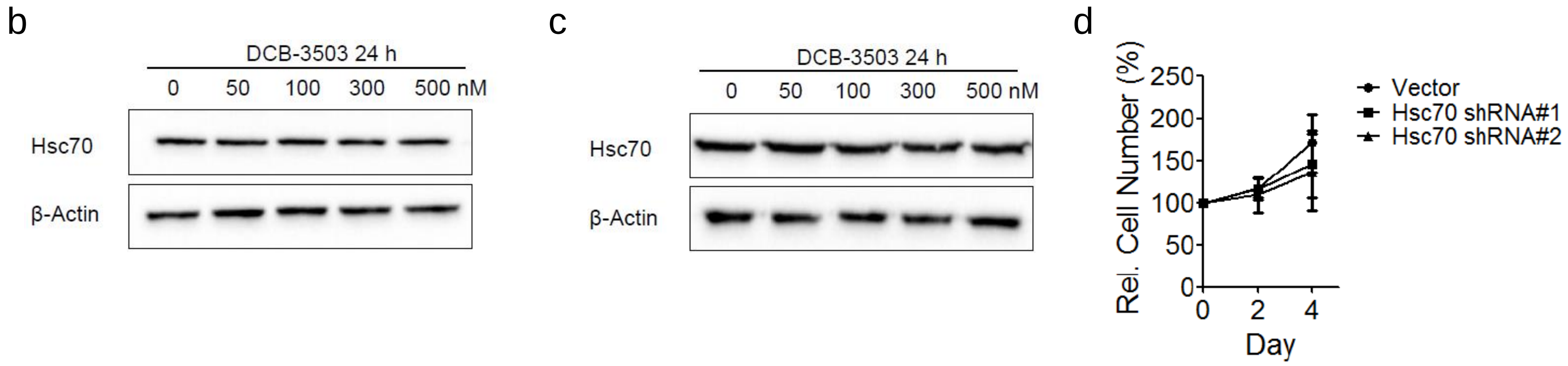

Figure S2

a

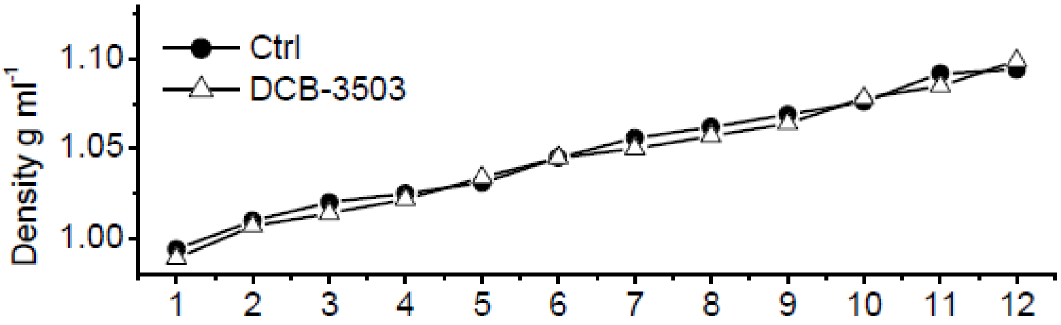

b

DCB-3503 300 nM	Input			Ctrl IP			HSC70 IP		
	0	1	4	0	1	4	0	1	4 h
Cyclin D1	1.00	0.83	1.73	0.15	0.13	0.12	0.45	0.87	0.89
Cyclin D2	1.00	0.79	1.63	0.1	0.15	0.16	0.4	0.73	0.83
Cyclin D3	1.00	0.93	0.84	0.05	0.07	0.08	0.35	0.49	0.51
β-Catenin	1.00	1.16	0.73	0.08	0.06	0.06	0.30	0.58	0.53
p21	1.00	1.16	0.99	0.14	0.07	0.04	0.33	0.32	0.18
p53	1.00	1.28	0.95	0.11	0.13	0.05	0.16	0.17	0.17
Survivin	1.00	0.87	0.45	0.04	0.04	0.02	0.03	0.05	0.02
Cyclin E1	1.00	1.03	0.8	0.04	0.08	0.04	0.12	0.17	0.16
β-Actin	1.00	1.18	0.84	0.02	0.02	0.02	0.14	0.09	0.12

Color scale: 2.00, 1.67, 1.33, 1.00, 0.67, 0.33, 0.00

c

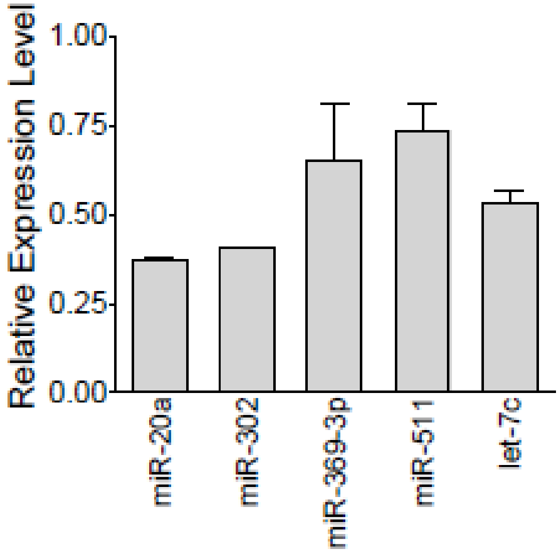

Figure S3

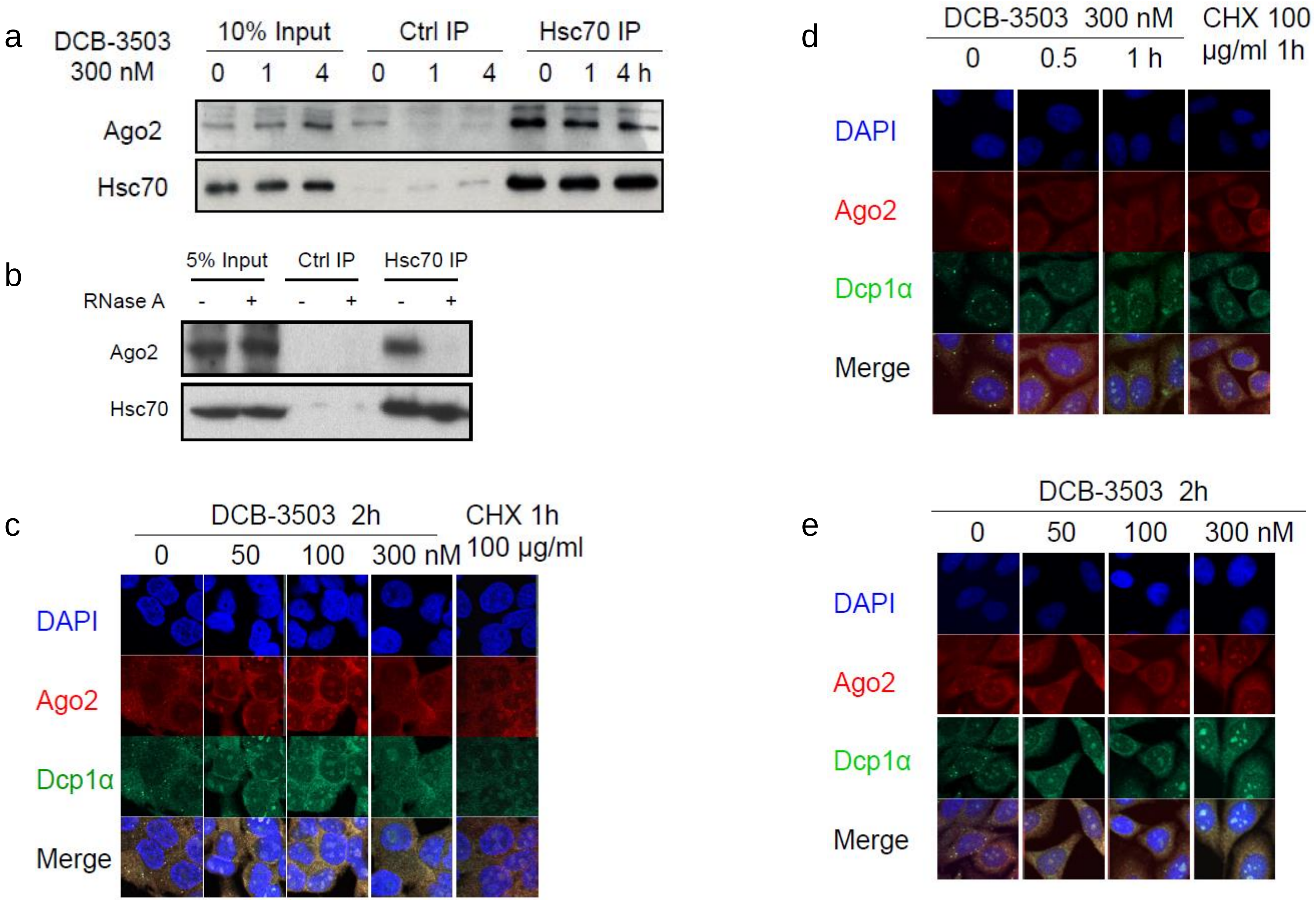

a
DCB-3503 300 nM
10% Input
Ctrl IP
Hsc70 IP
0 1 4
0 1 4
0 1 4 h
Ago2
Hsc70
b
5% Input
Ctrl IP
Hsc70 IP
RNase A - + - + - +
Ago2
Hsc70
c
DCB-3503 2h
0 50 100 300 nM
CHX 1h 100 μg/ml
DAPI
Ago2
Dcp1α
Merge
d
DCB-3503 300 nM
0 0.5 1 h
CHX 100 μg/ml 1h
DAPI
Ago2
Dcp1α
Merge
e
DCB-3503 2h
0 50 100 300 nM
DAPI
Ago2
Dcp1α
Merge
